# Immune-related biomarker risk score predicts prognosis in prostate cancer

**DOI:** 10.18632/aging.103921

**Published:** 2020-11-10

**Authors:** Zezhen Liu, Jiehui Zhong, Chao Cai, Jianming Lu, Wenqi Wu, Guohua Zeng

**Affiliations:** 1 Department of Urology, Minimally Invasive Surgery Center, Guangdong Key Laboratory of Urology, Guangzhou Urology Research Institute, The First Affiliated Hospital of Guangzhou Medical University, Guangzhou, Guangdong, China; 2 Guangdong Key Laboratory of Clinical Molecular Medicine and Diagnostics, Department of Urology, Guangzhou First People's Hospital, School of Medicine, South China University of Technology, Guangzhou, Guangdong, China

**Keywords:** prostate cancer, PLK1, immune-related genes, immune microenvironment

## Abstract

In this study, we constructed a model using a Cox proportional hazards model based on the expression of eight immune-related genes that were associated with prognosis in prostate cancer: EDNRB, ANGPTL2, TNFSF15, TNFRSF10D, EDN2, BMP2, NLRP14, and PLK1. We then identified associations between risk scores calculated with the model, tumor microenvironment characteristics, and immune cell infiltration. Prostate cancer patients in the high score group had poorer prognoses, and validation with the external GSE54460 dataset confirmed that the scoring model predicted biochemical recurrence with AUC values of 0.749 at 1 year, 0.804 at 3 years, and 0.774 at 5 years. Proportions of infiltrated M2 macrophages and regulatory T cells were increased in the high risk group, while CD8^+^ T cells were increased in the low risk group. Network analysis revealed that PLK1 may be a key regulator of the immune-suppressive microenvironment in prostate cancer. Double immunofluorescence labeling of a prostate cancer tissue microarray indicated that PLK1 expression correlated positively with numbers of infiltrating macrophages. These results indicate that an immune- related, gene-based risk score effectively reflects immune microenvironment characteristics and predicts prognosis in prostate cancer.

## INTRODUCTION

Prostate cancer poses a serious threat to the health of men all over the world [1, 2]. Although radical surgical excision and radiation therapy are effective in treating prostate cancer, only lung cancer has a higher mortality rate. In China, where prostate cancer screening is relatively uncommon, most patients have locally advanced or metastatic prostate cancer upon diagnosis. In these cases, endocrine therapy is typically used as the primary treatment. After about 18 months of endocrine therapy, most prostate cancers become resistant to hormone treatments, and no other effective clinical treatments are currently available. Novel immunotherapies are an important development in tumor treatment, and immune checkpoint inhibitors may be particularly effective. However, recent phase II clinical trials indicate that immune check point inhibitors are only effective against specific types of prostate cancer, and the disease control rate does not exceed 20% [3, 4].

The development of resistance to immunotherapies is the main reason for their poor efficacy in treating prostate cancer. PD-L1^+^ or VISTA^+^ M2 macrophages are the major drivers of prostate immunotherapy resistance [5]. M1 macrophages activated by the classical pathway can induce tissue inflammation, and T cells activated by inflammation can effectively kill or suppress prostate cancer cells. In contrast, M2 macrophages with anti-inflammatory characteristics can promote tissue repair and immunosuppression. M2 macrophages exert protective effects in tumors by inhibiting tumor antigen presentation. This prevents T cells from recognizing tumor antigens and immune checkpoint inhibitors, which regulate T cell function, from effectively treating prostate cancer [6–8]. Additional causes of insensitivity to immunotherapy in prostate cancer remain poorly understood. Further explorations of the immune characteristics of prostate cancer are therefore needed.

**Figure 1:**
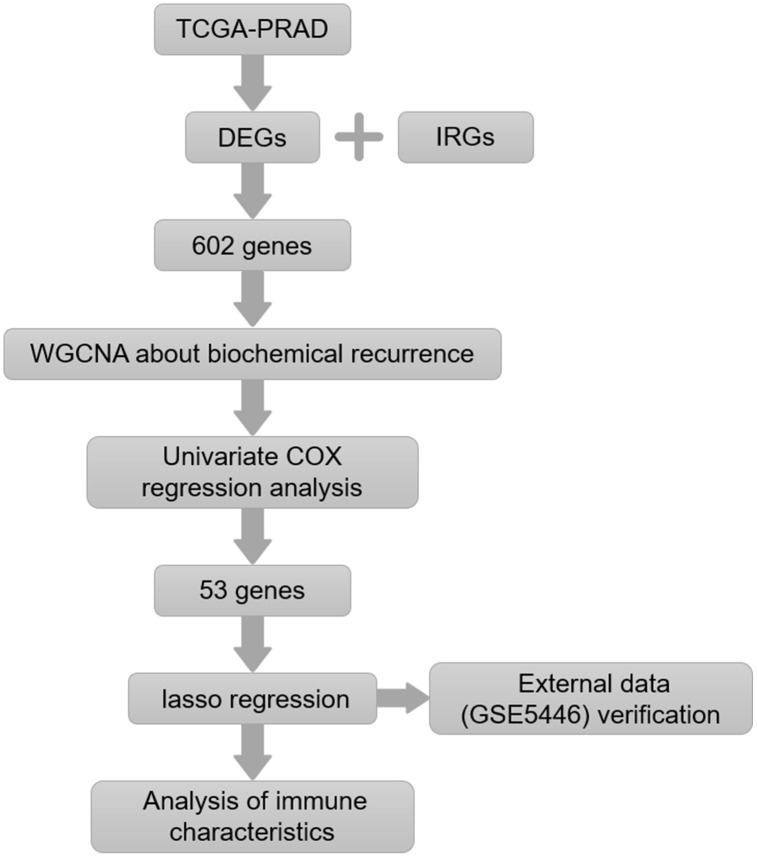
**Flow chart of the experimental strategy.**

Interactions between tumor cells and immune cells are complex, and tumor status can alter the tumor immune microenvironment. In this study, we examined relationships between gene expression and immune cell infiltration in prostate cancer. Immune genes associated with biochemical recurrence of prostate cancer were screened to construct a prognostic model. The effectiveness of the model was validated using an external dataset. Relationships between immune characteristic genes and immune cell infiltration in prostate cancer were analyzed. Finally, key genes that might induce crucial changes in the prostate cancer immune microenvironment were identified (Figure 1). The relationship between expression of these potential key genes and M2 macrophage infiltration was verified in tissue samples.

## RESULTS

### Weighted correlation network analysis (WGCNA) identified prostate cancer-related genes associated with biochemical recurrence (BCR)

**Figure 2:**
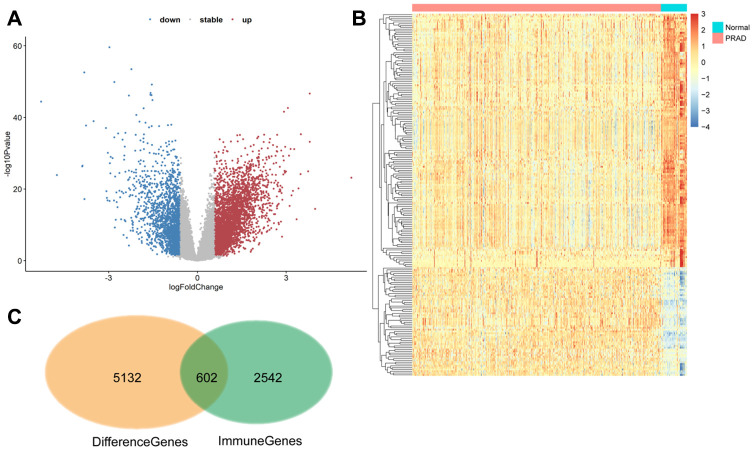
**602 differentially expressed immune-related genes were identified.** (**A** and **B**) Volcano plot and heat maps showing differentially expressed genes in TCGA prostate cancer samples. (**C**) The 602 differentially expressed immune genes were considered candidate genes for the risk model.

**Figure 3:**
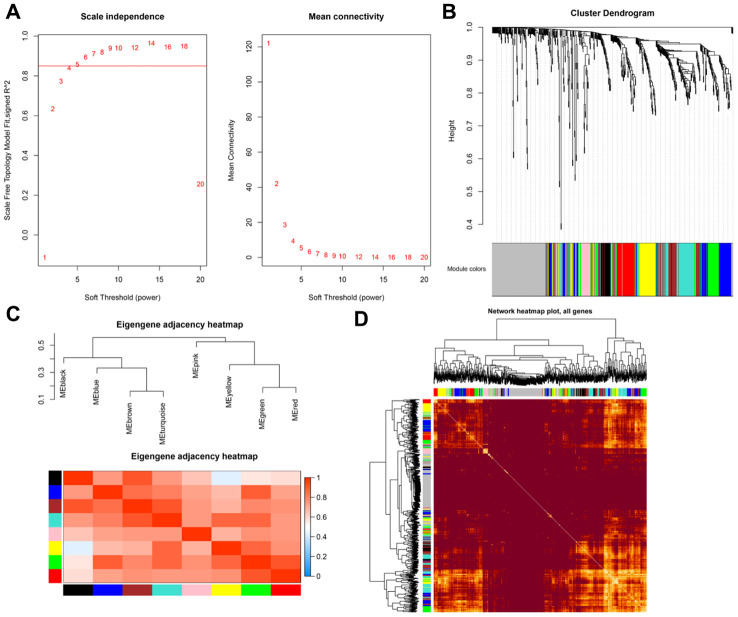
****(**A**) Scale-free fit index for soft-thresholding powers. (**B**) Dendrogram showing all differentially expressed genes clustered based on different metrics. (**C**) Heatmap of correlations between module eigengenes and clinical traits. (**D**) Visualization of gene networks.

The TCGA database includes 5132 genes that are differentially expressed in prostate cancer compared to normal prostate tissue. Among them, 602 were immune-regulated genes (IRGs). Weighted correlation network analysis (WGCNA) was performed using these 602 candidate genes based on TCGA PRAD cancer sample expression profiles (Figure 2). Eight samples with abnormal clustering were removed during the screening process. The soft-thresholding power in the WGCNA (β=5) was determined based on scale-free R2 > 0.85 (Figure 3A). Eight modules were identified by average linkage hierarchical clustering based the soft-thresholding power (Figure 3B). According to the hierarchical clustering of modules, there were significant correlations between most modules (Figure 3C). A network heatmap of all genes is shown in Figure 3D.

### Construction of an immunogenetic risk score associated with BCR in prostate cancer

**Figure 4:**
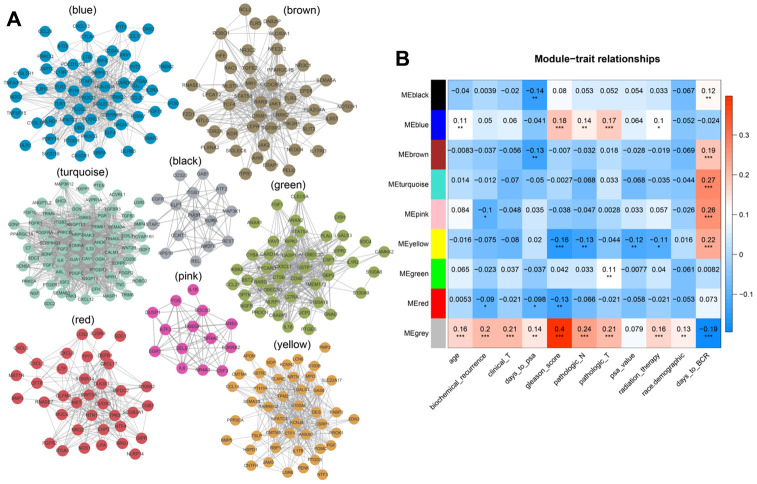
****(**A**) Modular genetic correlation network map. Colors correspond to different modules. (**B**) Correlations between modules and clinical phenotypes. Red indicates positive correlations, blue indicates negative correlations.

**Figure 5:**
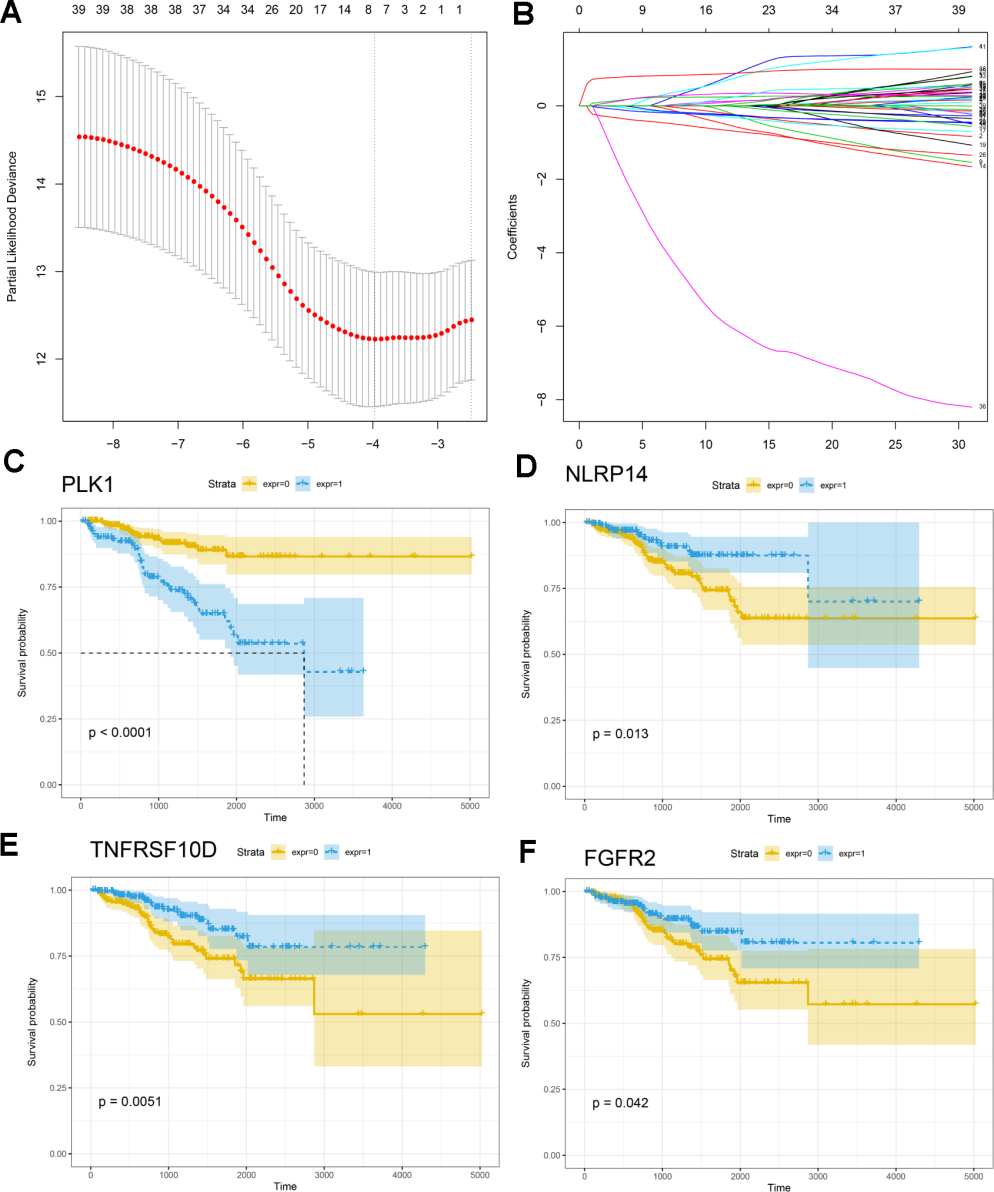
**Construction of the IRG-based prognostic model.** (**A**, **B**) The number of factors included in the model was determined through LASSO analysis. (**C**–**F**) KM curves for PLK1, NLRP14, TNFRSF10D, and FGFR2.

We analyzed associations between all modules and clinical characteristics of prostate cancer patients. The brown, turquoise, pink, and yellow modules were highly associated with time to BCR, and the pink and red modules were also associated with BCR (Figure 4). Further screening of the genes included in these modules revealed a correlation coefficients of greater than 0.1 between their expression and BCR in prostate cancer and of greater than 0.5 for gene expression correlations within the modules. In total, 221 immune-related genes were associated with BCR in prostate cancer (Supplementary Table 3). We then performed Cox univariate regression analysis to identify correlations between the above genes and BCR based on clinical information for prostate cancer patients in TCGA. A log-rank test revealed that 53 genes were associated with BCR in prostate cancer. Among these 53 genes, eight survival-associated IRGs (EDNRB, ANGPTL2, TNFSF15, TNFRSF10D, EDN2, BMP2, NLRP14, PLK1) were also identified using lasso regression analysis (Figure 5A, 5B). KM curves for PLK1, NLRP14, TNFRSF10D, and FGFR2 are shown in Figure 5C–5F; lasso regression coefficients for all eight IRGs are show in Table 1.

**Table 1:** Lasso regression.

**Symbol**	**Lasso regression coefficient**
DNRB	-0.057
ANGPTL2	0.168
TNFSF15	-0.007
TNFRSF10D	-0.357
EDN2	-0.123
BMP2	0.158
NLRP14	-1.952
PLK1	0.787

### Immunogenetic risk score is associated with BCR in prostate cancer

**Figure 6:**
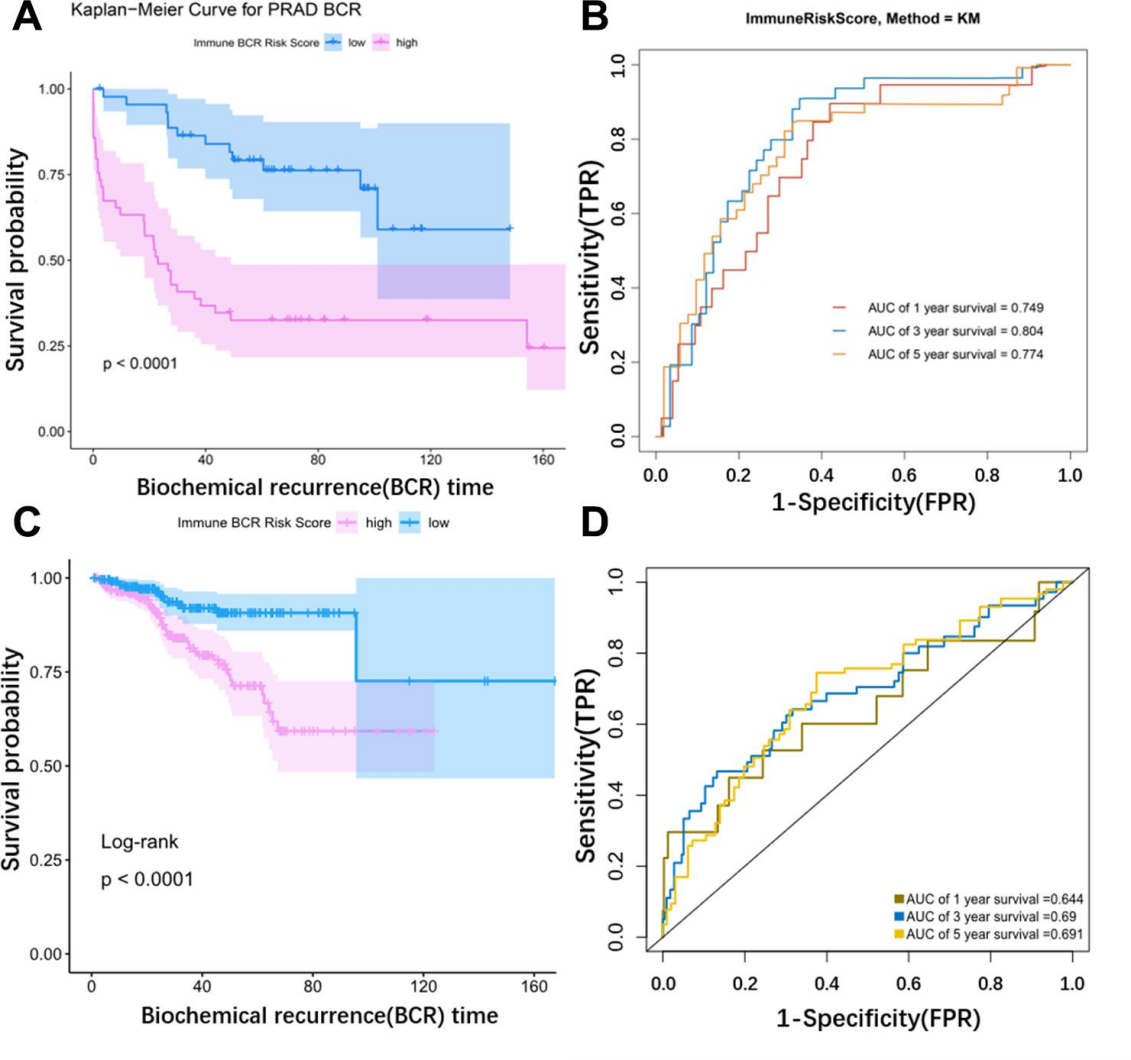
**Validation of the model using external data.** (**A**) KM curve for the external dataset (GSE54460). (**B**) Time dependent ROC curves. The AUC (Area Under Curve) was 0.749 at 1 year, 0.804 at 3 years, and 0.774 at 5 years in the GSE54460 cohort. (**C**) KM curve for TCGA. (**D**) Time dependent ROC curves. The AUC (Area Under Curve) was 0.644 at 1 year, 0.69 at 3 years, and 0.691 at 5 years in the TCGA cohort.

Risk scores for BCR in prostate cancer based on corresponding lasso coefficients were calculated for each sample in the training cohort (GSE54460). Risk score was significantly associated with BCR in prostate cancer patients; BCR occurred sooner in the high risk group (Figure 6A). An ROC curve was used to assess the effect of the Risk score. The AUC (Area Under Curve) was 0.749 at one year, 0.804 at 3 years, and 0.774 at 5 years in the testing cohort (Figure 6B). The ROC curve in the TCGA cohort was consistent with the curve in the training cohort (Figure 6C). The AUC was 0.644 at one year, 0.69 at 3 years, and 0.691 at 5 years in the TCGA cohort (Figure 6D). Risk score was therefore a useful predictor of BCR in prostate cancer. We also performed a multivariate correlation analysis to determine the impact of other clinical factors on the prognostic power of the risk score. Immune risk score was still associated with BCR in prostate cancer after multivariate adjustment (p=0.042) (Table 2).

**Table 2:** Multiple regression analysis was used to verify the model generated via lasso regression.

**Variate**	**Univ HR (95% CI for HR)**	**Univ p value**	**Multiv HR (95% CI for HR)**	**Multiv p value**
Age	1.0064 (0.9593-1.0559)	0.7938	0.9875 (0.9388-1.0388)	0.6265
Concentration (ng/μl)	1.0071 (1.0034-1.0107)	0.0001	0.9842 (0.9239-1.0484)	0.6207
Race (W VS B)	1.028 (0.4305-2.4546)	0.9505	1.2058 (0.4565-3.1853)	0.7057
Ratio 260/230	8.0448 (2.9966-21.5976)	<0.0001	4.9789 (0.7663-32.3485)	0.0927
Rpl13a Ct value	1.2157 (1.003-1.4734)	0.0465	1.1231 (0.8919-1.4142)	0.3234
Total yield (μg)	1.0909 (1.0402-1.1441)	0.0003	1.2078 (0.538-2.7116)	0.6472
Immune Risk score	7.2791 (2.3037-22.9999)	0.0007	3.7842 (1.0476-13.6691)	**0.0423**

### Differences in immune characteristics between high and low risk groups

**Figure 7:**
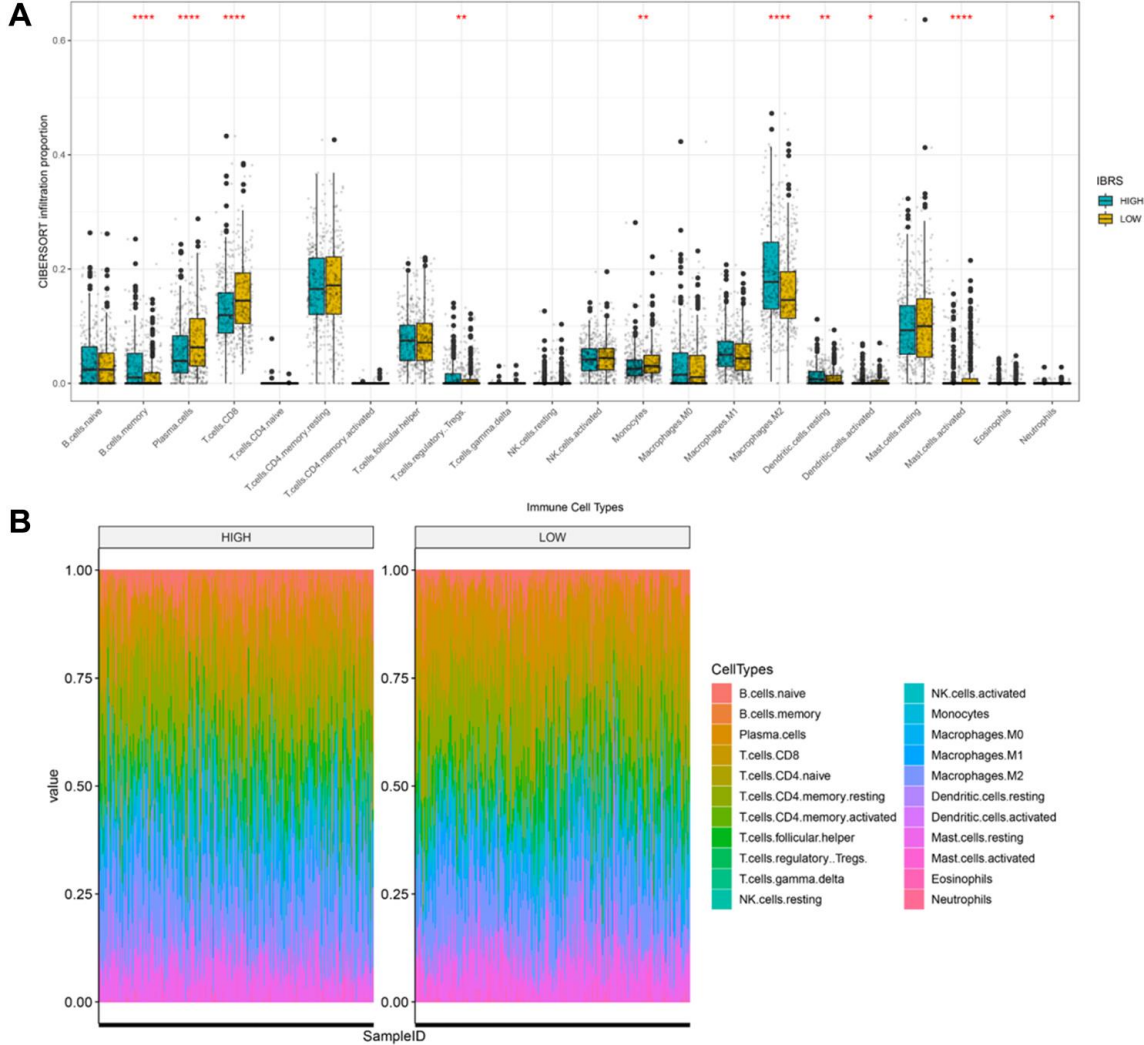
**Cibersort was used to calculate infiltration scores for 22 immune cell types based on the TCGA prostate cancer expression profile.** (**A**) Infiltration differences (ratio) in high and low risk groups. ****p<0.0001, ***p<0.001, **p<0.01, *p<0.05. (**B**) Infiltration profiles in the high and low risk groups.

**Figure 8:**
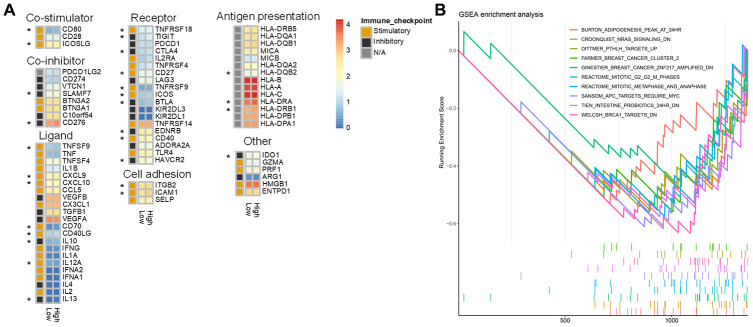
**Differences in immune characteristics between the high and low risk score groups.** (**A**) Expression of 75 immunomodulators in the high and low risk groups. “*” indicates a difference in expression between the high and low risk groups. (**B**) Immune-related GSEA enrichment analysis.

Next, we examined differences in the tumor microenvironment between low and high risk patients based on prostate cancer mRNA expression data from the TCGA database. Genes that were differentially expressed between the high and low risk score groups are shown in the volcano plot in Supplementary Figure 1. No significant differences in immune and stroma genes were observed between the high and low risk score groups (Supplementary Figure 2). However, differences were observed in infiltration levels for 22 types immune cells in CIBERSORT data. Memory B cell, regulatory T cell, M2 macrophage, and dendritic cell infiltration were higher in the high risk group, while plasma cell, CD8^+^T cell, monocyte, and activated mast cell infiltration were higher in the low score group (Figure 7A). Overall immune cell infiltration data for the high and low risk score groups is shown in Figure 7B. Expression of immunoregulatory factors in the tumor immune microenvironment also differed between the high and low risk groups. (Figure 8A); the 28 factors that differed significantly are shown in Supplementary Figure 3.

### Identification of pathways and gene ontology (GO) terms associated with high and low risk score groups

The ClusterProfiler R package was used to perform GSEA enrichment analysis on DEGs between the high and low score groups using gene sets from MsigDB as background genes. Log_2_(fold change) values from the differential expression analysis were used as the sorting criterion, and P value < 0.05 was used as the screening criterion (Supplementary Table 4). The results indicated that there were significant differences in “immune effector process,” “immune response,” “immune system process,” “innate immune response,” “positive regulation of immune system process,” “regulation of immune system process,” and “regulation of immune system process” between low and high risk groups (Figure 8B). GO enrichment analysis was also performed using p value < 0.05 and overlap > 0.75 as screening criteria (Supplementary Figure 4).

### PLK1 may be a key regulator of the immune microenvironment in prostate cancer

**Figure 9:**
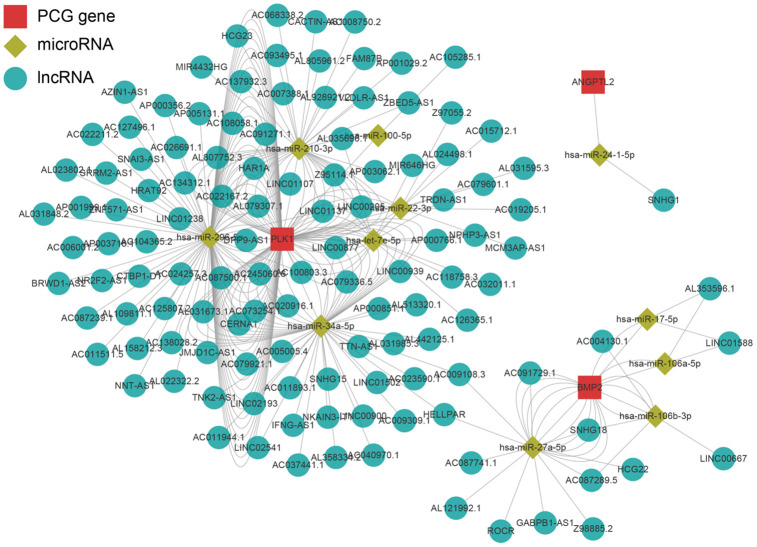
**Regulatory network of the key genes.** Red squares indicate key genes, green diamonds indicate microRNAs, and green circles indicate lncRNA. Key genes for which literature reporting validated regulatory networks was not available were omitted.

**Figure 10:**
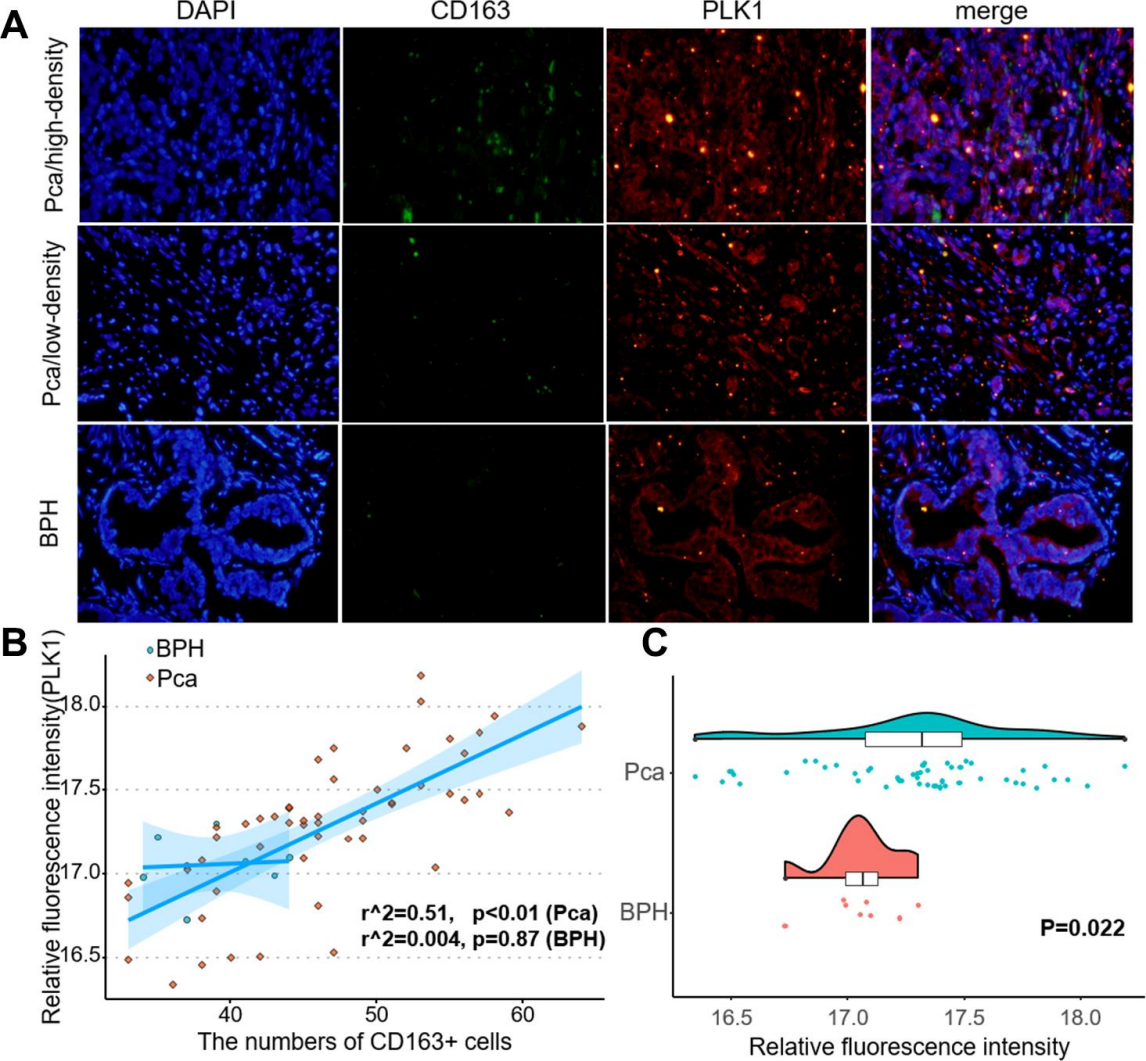
**PLK1 expression correlated positively with M2 macrophage infiltration.** (**A**) Fluorescence imaging of human prostate cancer and adjacent noncancerous tissues with FITC-labeled CD163 and Cy3-labeled PLK1. Most green fluorescent signals were observed on the cytomembrane, while red fluorescent signals were primarily located in the cytoplasm in prostate tissue. (**B**) Numbers of green fluorescent cells and red fluorescence integral optical density were positively correlated in prostate cancer samples (r^2^=0.51, p<0.01). (**C**) PLK1 staining was more intense in prostate cancer tissues than in non-cancerous prostate tissues.

The LnCeVar database contains transcription factor regulation data validated in the literature. As shown in Figure 9, PLK1 is part of a rich regulation system. In a previous study, we found that infiltration of M2 macrophages promotes prostate cancer progression. Here, risk score was also associated with M2 macrophages. Among the genes included in the risk score, PLK1 had the largest coefficient, indicating that it may be the most critical factor in the model. We therefore performed double immunofluorescence labeling of CD163, a marker of M2 macrophages, and PLK1 in a prostate cancer tissue microarray (Figure 10A). PLK1 was positively correlated with CD163 in prostate cancer (Pca) samples (r=0.69, p<0.01), but not in benign prostatic hypertrophy (BPH) samples (r=0.12, p=0.63) (Figure 10B). Furthermore, PLK1 expression was significantly increased in prostate cancer compared to prostatic hyperplasia. (p=0.022) (Figure 10C).

## DISCUSSION

Current treatments for prostate cancer include surgery, radiation therapy, and endocrine therapy. Recent studies indicate that the tumor immune microenvironment plays an important role in the development and progression of prostate cancer, and immunotherapy can provide significant benefits to some prostate cancer patients. However, regulatory T cells, tolerogenic dendritic cells, and non-functional T cells (CD4^+^ and CD8^+^) can induce immunosuppression in the tumor microenvironment [9]. Such immunosuppression is the main obstacle to the efficacy of immunotherapy-induced anti-tumor immune responses. Multi-drug combination therapy, which can increase the impact of immune checkpoint inhibitors by regulating the tumor microenvironment, is an important method for improving the efficacy of cancer immunotherapy [10]. In this study, we examined the immune characteristics of prostate cancer to identify potential key genes that regulate the tumor immune microenvironment.

We found that 53 immune-related genes were associated with biochemical recurrence in prostate cancer. Among these, eight were identified as key genes by lasso regression. Validation with an external dataset indicated that the prediction model was highly accurate (5 year AUC=0.774). Moreover, biochemical recurrence occurred sooner and survival rates were lower in the high risk score group. Analysis of the LnCeVar database indicated that PLK1 had a particularly rich regulatory system in prostate cancer among the eight key genes.

Decreased expression of EDNRB, TNFSF15, TNFRSF10D, EDN2, and NLRP14, as well as increased expression of ANGPTL2, BMP2, and PLK1, were associated with higher risk scores; EDNRB promoter methylation status was also associated with risk score. A meta-analysis of 11 similar studies indicated that the frequency of EDNRB methylation was substantially higher in prostate cancer compared with normal prostate tissues (OR = 5.42, 95 % CI = 1.98–14.88, P = 0.001), suggesting that EDNRB promoter methylation might increase the risk of prostate cancer [11]. Here, TNFSF15 expression was inversely associated with prostate cancer risk. This is consistent with the role of TNFSF15 as a downstream effector of AMPK that inhibits prostate cancer growth [12]. TNFRSF10D expression is also associated with prostate cancer and with the direct p53 effectors and ERK signaling pathways; here, TNFRSF10D was inversely related to prostate cancer biochemical recurrence risk score. Endothelins are involved in the regulation of various physiological processes, including plumage development in chickens, pigmentation, neural crest cell proliferation, differentiation, migration, cardiovascular development and functions, and pulmonary hypertension [13]. The endothelin EDN2 was inversely associated with prostate cancer immunological risk score in our model. As a member of a family of molecules that belong to a signal-induced multiprotein complex termed the inflammasome that activates proinflammatory caspase-1 and caspase-5, NLRP14 may play a regulatory role in the innate immune system. NLRP14 is considered an oncogene, and increased expression of NLRP14 is associated with increases in prostate cancer mortality [14]. This contradicts the inverse association found here between NLRP14 and risk score, and further study is warranted. ANGPTL2 is a secreted glycoprotein with homology to angiopoietins that may exert autocrine or paracrine effects on endothelial cells. ANGPTL2 also promotes M2 polarization of macrophages in non-small cell lung cancer [15]. In addition, ANGPTL2 may promote acquisition of androgen independence and tumor progression in prostate cancer by exerting autocrine and/or paracrine effects via the integrin α5β1 receptor [16]. Here, we found a positive association between ANGPTL2 expression and immune-related risk score in prostate cancer. Finally, several studies suggest that BMP2 promotes progression and induces biochemical recurrence in prostate cancer [17–19], which is consistent with the positive association found here between BMP2 and risk score.

Our present results demonstrate that memory B cell, regulatory T cell, M2 macrophage, and dendritic cell infiltration were significantly increased in the high risk score group, while plasma cell, CD8+T cell, monocyte, and activated mast cell infiltration were higher in the low risk score group. M2 macrophages promote prostate cancer progression and help establish an immunosuppressive state in tumors [20–21]; this might help explain the increased M2 macrophage infiltration observed in the higher risk score group. In contrast, local increases in the density of infiltrating CD8+T cells in tumors is a marker of good prognosis; this might account for the increased CD8+T cell infiltration observed here in the low risk score group with better prognoses. Infiltration of these cell types reflects the immune microenvironment and can also predict prognosis in prostate cancer [22].

The role of PLK1 in prostate cancer is not clear at present [23]. However, numerous studies indicate that PLK1 can act as an oncogene, and PLK-1 inhibitors can effectively inhibit prostate cancer progression [24–26]. PLK1 can also inactivate other tumor suppressors [27, 28]. Another study suggests that PLK1 is a carcinogenic factor [29]. Here, we found that PLK1 expression was higher in the high risk group that experienced earlier biochemical recurrence. Moreover, downstream pathways regulated by PLK1 comprised the single largest pathway group in our biochemical recurrence prediction model for prostate cancer. PLK1 might therefore be one of the most important immune genes that contribute to biochemical recurrence in prostate cancer.

## MATERIALS AND METHODS

### Data acquisition

The training dataset was obtained from TCGA and included 498 cancer samples and 52 normal control samples; clinical information for those samples is shown in Supplementary Table 1. The GSE54460 testing dataset containing 106 samples was downloaded from the gene expression omnibus database (GEO: https://www.ncbi.nlm.nih.gov/geo/); clinical information for those samples is shown in Supplementary Table 2. Immune genes that were included in our analyses were obtained from InnateDB (https://www.innatedb.ca/) and ImmPort (https://www.immport.org/home).

### Identification of differentially expressed genes

Genes that were differentially expressed between normal and prostate cancer samples were identified based on the screening criteria of p value > 0.05 and log(fold-change) > 1.5.

### Weighted gene co-expression network analysis

TCGA expression data for 602 immune-related genes was used for WGCNA (weighted gene co-expression network analysis) to identify associations between gene expression modules and clinical characteristics [30]. During sample clustering, eight samples with abnormal clustering were identified and removed from the analysis (Supplementary Figure 1). The co-expression network was then constructed and divided into modules. A scale-free network coefficient of greater than 0.85 was used to ensure that the co-expression network conformed to the scale-free network standards. Gene significance (GS) indicates the strength of linear correlations between the expression of different gene modules and clinical features. Modules with P≤0.01 and higher GS values were identified as survival-related modules and included in subsequent analysis.

### Univariate Cox regression and lasso regression

The Survival package for R was used to perform univariate Cox regression and the KM test. The Glmnet package was used for lasso regression. Prognosis related genes were screen and correlation regression coefficients were obtained. The resulting risk score was validated in the GEO dataset (GSE54460). “TimeROC” was used to draw receiver operating characteristic curves (ROC), and the area under the curve (AUC) was calculated. Samples were divided between the high and low risk groups using the median risk score (RS) as a cutoff value. KM curves were used to evaluate the survival of prostate cancer patients.

### Comparison of immune cell infiltration between high and low risk groups

The “ESTIMATE” package was used to calculate the microenvironment score, and the “CIBERSORT” package was used to assess the proportions of 22 leukocyte subtypes based on differences in mRNA expression between the high and low risk groups [31]. The Wilcox test was used to evaluate differences between the high low risk score groups.

### Double immunofluorescence

Tissue microarrays (DC-Pro11018, Xian, China) including 74 prostate cancer tissues and 6 non-cancer prostate tissues, along with associated detailed clinical information, were purchased from Alenabio Biotech (Xian, China). Clinical information for the prostate cancer tissue microarray is shown in Supplementary Table 5. The sections were incubated overnight at 4°C with antibodies against anti-CD163 (rabbit, 1:100, A8383, Elabscience, Wuhan, China) and anti-PLK1 (mouse, 1:50, TA500393S, ORIGENE, Rockville, USA). The sections were then washed three times with cold PBS and stained with Cy3 Goat Anti-Mouse IgG (H+L) (1:100, AS008, ABclonal, Wuhan, China) or FITC Goat Anti-Rabbit IgG (H+L) (1:100, AS011, AS008, ABclonal, Wuhan, China) secondary antibodies. Nuclei were stained with DAPI. Stained tissues were visualized using an Olympus IX73 microscope (Waltham, MA). IOD (integral optical density) was calculated using ImageJ (1.46r, National Institutes of Health, USA).

### Statistical analysis

All statistical analyses were performed using R software (version 3.6.3, http://www.R-project.org). A two-sided P < 0.05 indicated a statistically significant difference.

## Supplementary Material

Supplementary Figures

Supplementary Tables 1 and 2

Supplementary Table 3

Supplementary Table 4

Supplementary Table 5

## References

[1] Siegel RL, Miller KD, Jemal A. Cancer statistics, 2020. CA Cancer J Clin. 2020; 70:7–30. 10.3322/caac.21590 31912902

[2] Bray F, Ferlay J, Soerjomataram I, Siegel RL, Torre LA, Jemal A. Global cancer statistics 2018: GLOBOCAN estimates of incidence and mortality worldwide for 36 cancers in 185 countries. CA Cancer J Clin. 2018; 68:394–424. 10.3322/caac.21492 30207593

[3] Jindal V. Immunotherapy: a glimmer of hope for metastatic prostate cancer. Chin Clin Oncol. 2018; 7:61. 10.21037/cco.2018.02.01 29860848

[4] Antonarakis ES, Piulats JM, Gross-Goupil M, Goh J, Ojamaa K, Hoimes CJ, Vaishampayan U, Berger R, Sezer A, Alanko T, de Wit R, Li C, Omlin A, et al. Pembrolizumab for treatment-refractory metastatic castration-resistant prostate cancer: multicohort, open-label phase II KEYNOTE-199 study. J Clin Oncol. 2020; 38:395–405. 10.1200/JCO.19.01638 31774688 PMC7186583

[5] Gao J, Ward JF, Pettaway CA, Shi LZ, Subudhi SK, Vence LM, Zhao H, Chen J, Chen H, Efstathiou E, Troncoso P, Allison JP, Logothetis CJ, et al. VISTA is an inhibitory immune checkpoint that is increased after ipilimumab therapy in patients with prostate cancer. Nat Med. 2017; 23:551–55. 10.1038/nm.4308 28346412 PMC5466900

[6] Uderhardt S, Martins AJ, Tsang JS, Lämmermann T, Germain RN. Resident macrophages cloak tissue microlesions to prevent neutrophil-driven inflammatory damage. Cell. 2019; 177:541–55.e17. 10.1016/j.cell.2019.02.028 30955887 PMC6474841

[7] El-Kenawi A, Gatenbee C, Robertson-Tessi M, Bravo R, Dhillon J, Balagurunathan Y, Berglund A, Vishvakarma N, Ibrahim-Hashim A, Choi J, Luddy K, Gatenby R, Pilon-Thomas S, et al. Acidity promotes tumour progression by altering macrophage phenotype in prostate cancer. Br J Cancer. 2019; 121:556–566. 10.1038/s41416-019-0542-2 31417189 PMC6889319

[8] Kowal J, Kornete M, Joyce JA. Re-education of macrophages as a therapeutic strategy in cancer. Immunotherapy. 2019; 11:677–89. 10.2217/imt-2018-0156 31088236

[9] Jafari S, Molavi O, Kahroba H, Hejazi MS, Maleki-Dizaji N, Barghi S, Kiaie SH, Jadidi-Niaragh F. Clinical application of immune checkpoints in targeted immunotherapy of prostate cancer. Cell Mol Life Sci. 2020; 77:3693–3710. 10.1007/s00018-020-03459-1 32006051 PMC11104895

[10] Boettcher AN, Usman A, Morgans A, VanderWeele DJ, Sosman J, Wu JD. Past, current, and future of immunotherapies for prostate cancer. Front Oncol. 2019; 9:884. 10.3389/fonc.2019.00884 31572678 PMC6749031

[11] Yuan Y, Du Y, Wang L, Liu X. The value of endothelin receptor type B promoter methylation as a biomarker for the risk assessment and diagnosis of prostate cancer: a meta-analysis. Pathol Res Pract. 2020; 216:152796. 10.1016/j.prp.2019.152796 31926772

[12] Zhou J, Yang Z, Tsuji T, Gong J, Xie J, Chen C, Li W, Amar S, Luo Z. LITAF and TNFSF15, two downstream targets of AMPK, exert inhibitory effects on tumor growth. Oncogene. 2011; 30:1892–900. 10.1038/onc.2010.575 21217782 PMC3431012

[13] Ling L, Maguire JJ, Davenport AP. Endothelin-2, the forgotten isoform: emerging role in the cardiovascular system, ovarian development, immunology and cancer. Br J Pharmacol. 2013; 168:283–95. 10.1111/j.1476-5381.2011.01786.x 22118774 PMC3572556

[14] Wang H, Shen L, Li Y, Lv J. Integrated characterisation of cancer genes identifies key molecular biomarkers in stomach adenocarcinoma. J Clin Pathol. 2020; 73:579–586. 10.1136/jclinpath-2019-206400 32034058 PMC7476269

[15] Wei X, Nie S, Liu H, Sun J, Liu J, Li J, Li S, Wang S, Han S, Wang J, Sun Y. Angiopoietin-like protein 2 facilitates non-small cell lung cancer progression by promoting the polarization of M2 tumor-associated macrophages. Am J Cancer Res. 2017; 7:2220–33. 29218246 PMC5714751

[16] Sato R, Yamasaki M, Hirai K, Matsubara T, Nomura T, Sato F, Mimata H. Angiopoietin-like protein 2 induces androgen-independent and Malignant behavior in human prostate cancer cells. Oncol Rep. 2015; 33:58–66. 10.3892/or.2014.3586 25370833 PMC4254678

[17] Lai TH, Fong YC, Fu WM, Yang RS, Tang CH. Osteoblasts-derived BMP-2 enhances the motility of prostate cancer cells via activation of integrins. Prostate. 2008; 68:1341–53. 10.1002/pros.20799 18512729

[18] Horvath LG, Henshall SM, Kench JG, Turner JJ, Golovsky D, Brenner PC, O’Neill GF, Kooner R, Stricker PD, Grygiel JJ, Sutherland RL. Loss of BMP2, Smad8, and Smad4 expression in prostate cancer progression. Prostate. 2004; 59:234–42. 10.1002/pros.10361 15042598

[19] Tae BS, Cho S, Kim HC, Kim CH, Kang SH, Lee JG, Kim JJ, Park HS, Cheon J, Oh MM, Kang SG. Decreased expression of bone morphogenetic protein-2 is correlated with biochemical recurrence in prostate cancer: Immunohistochemical analysis. Sci Rep. 2018; 8:10748. 10.1038/s41598-018-28566-9 30013089 PMC6048060

[20] Zheng T, Ma G, Tang M, Li Z, Xu R. IL-8 secreted from M2 macrophages promoted prostate tumorigenesis via STAT3/MALAT1 pathway. Int J Mol Sci. 2018; 20:98. 10.3390/ijms20010098 30591689 PMC6337597

[21] Liu ZZ, Han ZD, Liang YK, Chen JX, Wan S, Zhuo YJ, Cai ZD, Deng YL, Lin ZY, Mo RJ, He HC, Zhong WD. TRIB1 induces macrophages to M2 phenotype by inhibiting IKB-zeta in prostate cancer. Cell Signal. 2019; 59:152–62. 10.1016/j.cellsig.2019.03.017 30926388

[22] Evans JD, Morris LK, Zhang H, Cao S, Liu X, Mara KC, Stish BJ, Davis BJ, Mansfield AS, Dronca RS, Iott MJ, Kwon ED, Foote RL, et al. Prospective immunophenotyping of CD8^+^ T cells and associated clinical outcomes of patients with oligometastatic prostate cancer treated with metastasis-directed SBRT. Int J Radiat Oncol Biol Phys. 2019; 103:229–40. 10.1016/j.ijrobp.2018.09.001 30205124 PMC6301146

[23] de Cárcer G. The Mitotic Cancer Target Polo-Like Kinase 1: Oncogene or Tumor Suppressor? Genes (Basel). 2019; 10:208. 10.3390/genes10030208 30862113 PMC6470689

[24] Li J, Wang R, Kong Y, Broman MM, Carlock C, Chen L, Li Z, Farah E, Ratliff TL, Liu X. Targeting Plk1 to enhance efficacy of olaparib in castration-resistant prostate cancer. Mol Cancer Ther. 2017; 16:469–79. 10.1158/1535-7163.MCT-16-0361 28069876 PMC5337144

[25] Shao C, Ahmad N, Hodges K, Kuang S, Ratliff T, Liu X. Inhibition of polo-like kinase 1 (Plk1) enhances the antineoplastic activity of metformin in prostate cancer. J Biol Chem. 2015; 290:2024–33. 10.1074/jbc.M114.596817 25505174 PMC4303657

[26] Shin SB, Woo SU, Yim H. Cotargeting Plk1 and androgen receptor enhances the therapeutic sensitivity of paclitaxel-resistant prostate cancer. Ther Adv Med Oncol. 2019; 11:1758835919846375. 10.1177/1758835919846375 31156720 PMC6515847

[27] Eckerdt F, Strebhardt K. Polo-like kinase 1: target and regulator of anaphase-promoting complex/cyclosome-dependent proteolysis. Cancer Res. 2006; 66:6895–98. 10.1158/0008-5472.CAN-06-0358 16849530

[28] Seki A, Coppinger JA, Du H, Jang CY, Yates JR 3rd, Fang G. Plk1- and beta-TrCP-dependent degradation of bora controls mitotic progression. J Cell Biol. 2008; 181:65–78. 10.1083/jcb.200712027 18378770 PMC2287288

[29] Simizu S, Osada H. Mutations in the plk gene lead to instability of plk protein in human tumour cell lines. Nat Cell Biol. 2000; 2:852–54. 10.1038/35041102 11056542

[30] Langfelder P, Horvath S. WGCNA: an R package for weighted correlation network analysis. BMC Bioinformatics. 2008; 9:559. 10.1186/1471-2105-9-559 19114008 PMC2631488

[31] Newman AM, Steen CB, Liu CL, Gentles AJ, Chaudhuri AA, Scherer F, Khodadoust MS, Esfahani MS, Luca BA, Steiner D, Diehn M, Alizadeh AA. Determining cell type abundance and expression from bulk tissues with digital cytometry. Nat Biotechnol. 2019; 37:773–82. 10.1038/s41587-019-0114-2 31061481 PMC6610714

