# Supplementary material for: Immune-related biomarker risk score predicts prognosis in prostate cancer
Source: Aging (Albany NY). 2020 Nov 10;12(22):22776–93. doi: 10.18632/aging.103921 (PMC7746334; doi:10.18632/aging.103921)
Supplement: Supplementary Figures [file aging-12-103921-s001..pdf]

SUPPLEMENTARY FIGURES

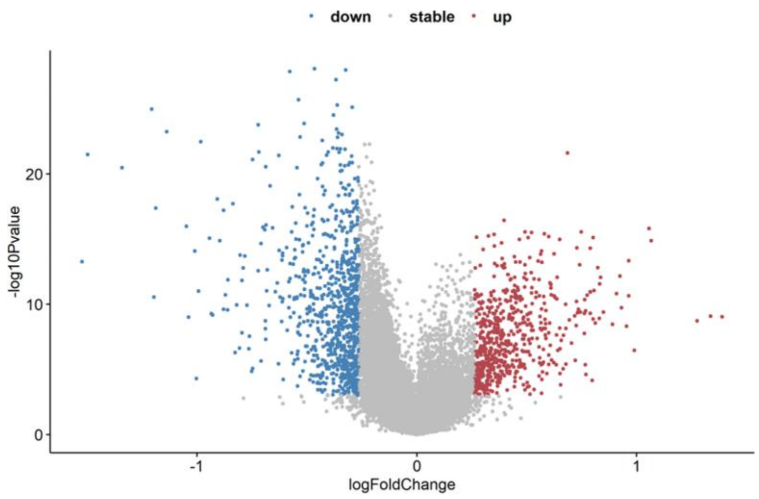

Supplementary Figure 1. Volcano map showing genes that were differential expressed between the high and low risk score groups. Red dots represent genes that are up-regulated in the high risk group, and blue dots represent genes that are down-regulated in the high risk group.

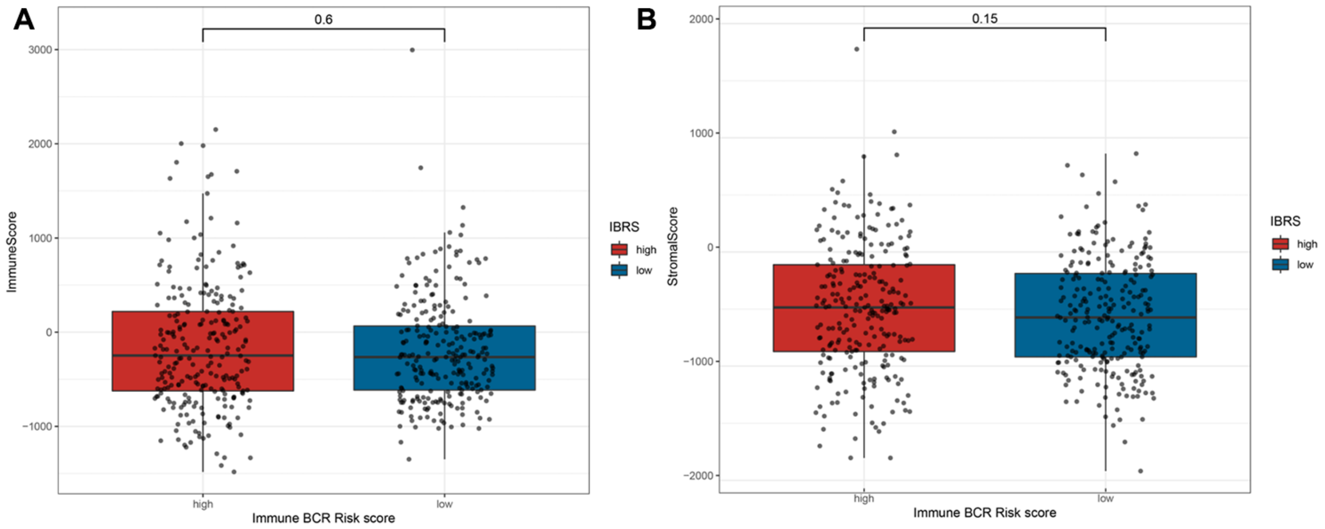

Supplementary Figure 2. (**A**) Immune scores and (**B**) matrix scores in the high and low risk groups.

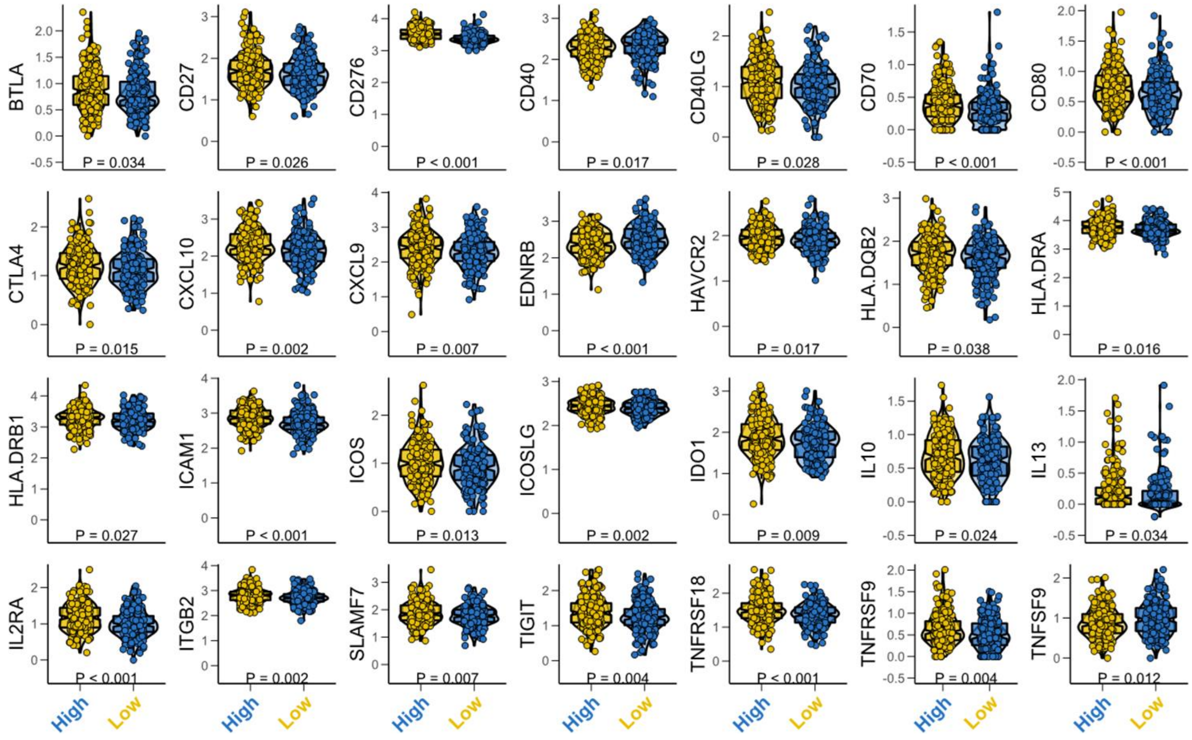

Supplementary Figure 3. 28 immunomodulatory factors differentially expressed between the high and low risk score groups.

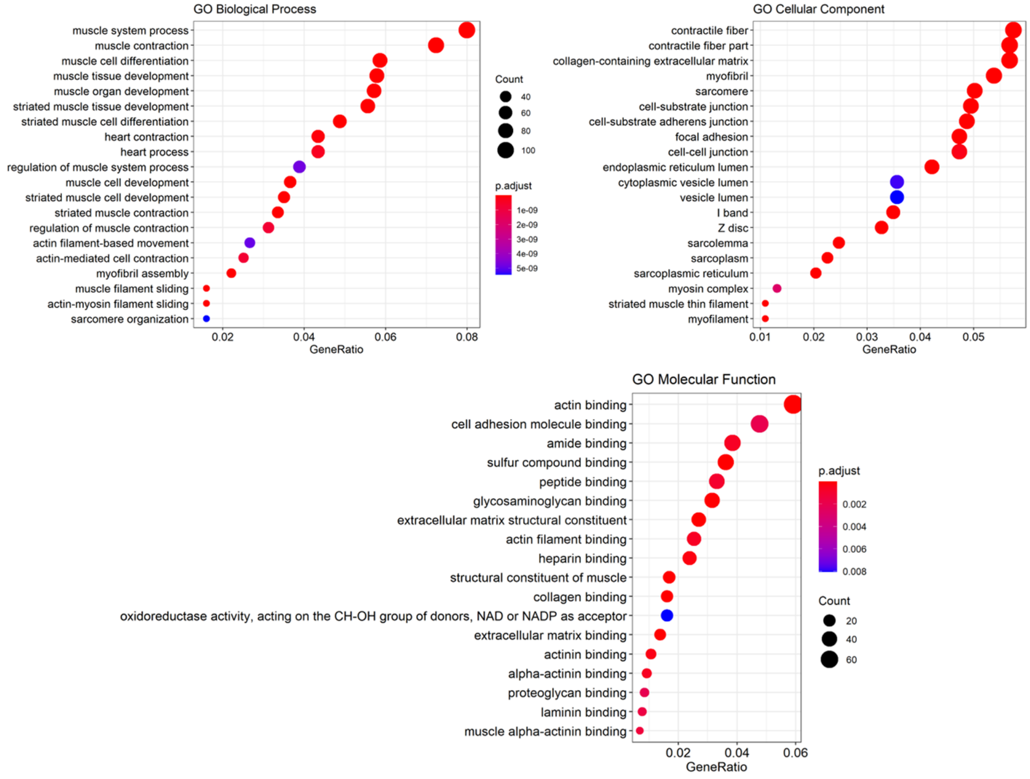

Supplementary Figure 4. GO enrich analysis was performed for differentially expressed genes.
