# Supplementary material for: Immune-related biomarker risk score predicts prognosis in prostate cancer
Source: Aging (Albany NY). 2020 Nov 10;12(22):22776–93. doi: 10.18632/aging.103921 (PMC7746334; doi:10.18632/aging.103921)
Supplement: Supplementary Tables 1 and 2 [file aging-12-103921-s002..pdf]

SUPPLEMENTARY TABLES

Supplementary Table 1. Clinical information of TCGA prostate cancer samples.

Characteristics		Counts or percent
Age	median(range)	61(41-78)
Biochemical_recurrence(BCR)		
	Yes	68(12.8%)
	No	464(87.2%)
Days_to_BCR	median(range)	708.0(58.0-2459.0)
Days_to_last_follow_up	median(range)	958(23-5024)
Clinical_T		
	T1a	1(0.2%)
	T1b	2(0.4%)
	T1c	212(42.1%)
	T2	13(2.6%)
	T2a	72(14.3%)
	T2b	72(14.3%)
	T2c	62(12.3%)
	T3a	48(9.5%)
	T3b	19(3.8%)
	T4	2(0.4%)
Days_to_psa	median(range)	518.5(-164.0 - 3447.0)
Gleason_score	median(range)	7(6-10)
Pathologic_N		
	N0	442(82.2%)
	N1	96(17.8%)
Pathologic_T		
	T2a	14(2.3%)
	T2b	12(1.9%)
	T2c	211(34.3%)
	T3a	203(33.0%)
	T3b	162(26.3%)
	T4	14(2.3%)
Psa_value	median(range)	0.1(0-323)
Radiation_therapy		
	NO	448(86.8%)
	YES	68(13.2%)
Race.demographic		
	american indian or alaska native	1(0.16%)
	asian	13(2.09%)
	black or african american	81(13.00%)
	not reported	18(2.89%)
	white	510(81.9%)

Supplementary Table 2. Clinical information of GSE54460 prostate cancer samples.

Characteristics		Counts or percent
Age	median(range)	61.7(43-78)
Concentration(ng.µl)	median(range)	95.39(17.04-757.53)
race		
	Black	22(31.4%)
	White	48(68.6%)
Ratio(260/230)	median(range)	0.87(0.16-1.86)
Rpl13a.Ct.value	median(range)	28.61(24.50-30.90)
Total yield(µg)	median(range)	6.55(1.50-25.36)
BCR		
	0	51(48.1%)
	1	55(51.8%)
months to BCR	median(range)	21.40(0-154.22)
months to lastFollow	median(range)	68.50(0.70-180.56)
