# Supplementary material for: Immune-related biomarker risk score predicts prognosis in prostate cancer
Source: Aging (Albany NY). 2020 Nov 10;12(22):22776–93. doi: 10.18632/aging.103921 (PMC7746334; doi:10.18632/aging.103921)
Supplement: Supplementary Table 5 [file aging-12-103921-s005..pdf]

Supplementary Table 5. Clinical information of tissue microarray of prostate cancer.

Characteristics	Counts or mean
**Age** (years)	68.29±10.20
**T classification** (n)	
T1	2
T2	49
T3	20
T4	2
**N classification**	
N0	66
N1	7
**M classification**	
M0	69
M1	4
**Grade**	
1	4
2	18
2-3	2
3	38
unknown	11
**Stage**	
I	3
II	43
IIB	1
III	15
IIIB	1
IV	10
